# miR-141-3p accelerates ovarian cancer progression and promotes M2-like macrophage polarization by targeting the Keap1-Nrf2 pathway

**DOI:** 10.1515/med-2023-0729

**Published:** 2023-06-09

**Authors:** Jingyun Zhao, Leilei Liu, Wei Zhao, Cuiting Lv, Na Zhang, Xinzhuan Jia, Zhengmao Zhang

**Affiliations:** Department of Reproductive Medicine, The Fourth Hospital of Hebei Medical University, Shijiazhuang, 050011, Hebei, China; Department of Obstetrics, The Fourth Hospital of Hebei Medical University, Shijiazhuang, 050011, Hebei, China; Department of Gynecology, The Fourth Hospital of Hebei Medical University, Shijiazhuang, 050011, Hebei, China; Department of Gynecology, The Fourth Hospital of Hebei Medical University, No. 12 Jiankang Road, Shijiazhuang, 050011, Hebei, China

**Keywords:** ovarian cancer, macrophage, polarization, miR-141-3p, Keap1, Nrf2

## Abstract

The miR-141-3p has been reported to participate in regulating autophagy and tumor-stroma interactions in ovarian cancer (OC). We aim to investigate whether miR-141-3p accelerates the progression of OC and its effect on macrophage 2 polarization by targeting the Kelch-like ECH-associated protein1-Nuclear factor E2-related factor2 (Keap1-Nrf2) pathway. SKOV3 and A2780 cells were transfected with miR-141-3p inhibitor and negative control to confirm the regulation of miR-141-3p on OC development. Moreover, the growth of tumors in xenograft nude mice treated by cells transfected with miR-141-3p inhibitor was established to further testify the role of miR-141-3p in OC. The expression of miR-141-3p was higher in OC tissue compared with non-cancerous tissue. Downregulation of miR-141-3p inhibited the proliferation, migration, and invasion of ovarian cells. Furthermore, miR-141-3p inhibition also suppressed M2-like macrophage polarization and *in vivo* OC progression. Inhibition of miR-141-3p significantly enhanced the expression of Keap1, the target gene of miR-141-3p, and thus downregulated Nrf2, while activation of Nrf2 reversed the reduction in M2 polarization by miR-141-3p inhibitor. Collectively, miR-141-3p contributes to tumor progression, migration, and M2 polarization of OC by activating the Keap1-Nrf2 pathway. Inhibition of miR-141-3p attenuates the malignant biological behavior of ovarian cells by inactivating the Keap1-Nrf2 pathway.

## 1 Introduction

Ovarian cancer (OC) is a common cause of morbidity and mortality in women with early metastasis and high malignancy [1]. Currently, the incidence and progression of OC are related to a variety of factors, including gene mutation and signal pathway abnormalities [2]. With the progress of basic and clinical research, people have a deeper understanding of the development and progress of OC, but the survival and prognosis of OC patients have not improved significantly in recent decades. Therefore, the treatment of patients with OC remains a major clinical challenge, requiring further research on tumorigenesis and new therapies at the molecular level.

MicroRNAs (miRNAs) are small endogenous non-coding RNA molecules consisting of approximately 21–25 nucleotides. These small miRNAs typically target one or more mRNAs, and they regulate gene expression by inhibiting or disrupting target mRNAs at the translational level. miRNAs are abnormally expressed in a variety of malignant tumors and closely related to the occurrence and development of cancers [3,4]. Studies have shown that miR-141-3p is involved in the occurrence and development of a variety of tumors including colorectal cancer [5], prostate cancer [6], breast cancer [7], and so on. At present, the research on the roles of miR-141-3p in OC is limited, and the conclusions are inconsistent. Therefore, our study will first detect the expression of miR-141-3p in ovarian carcinoma tissues and further investigate its molecular mechanisms.

The tumor microenvironment mainly consisted of the extracellular matrix, fibroblasts, vascular endothelial cells, and immunocytes [8]. These components play important roles in tumor structure and biology, contributing to tumor cells’ survival, proliferation, and migration [9]. Macrophages are attracted and activated by tumor microenvironment-derived cytokines, further differentiating into tumor-associated macrophages (TAMs) and contributing to complicated inflammation conditions [10]. It has indicated that TAMs were multifunctional and heterogeneous and will differentiate into M2-like macrophage, which exerts anti-inflammatory and expresses specific series of molecules, including Arginase 1 (Arg1), interleukin 10 (IL-10), and C-C Motif Chemokine Receptor 2 (CCR2) [11]. In contrary, M1-like macrophage is pro-inflammatory and secretes Il-1β and TNF-α, which prevent the cancerous phenotype. Therefore, M2 was highly associated with worse clinical prognosis [12] and higher density of M2 TAMs appears to correlate with tumor cell proliferation, metastasis, and intra-tumoral microvascular density, and M1 generally indicates better prognosis [13].

Nuclear factor E2-related factor2 (Nrf2), also known as carotenoid 2-like factor2 (NFE2L2), belong to the CNC-bZIP (cap “n” collar/basic Leucine zipper) family of transcription factors [12,14]. Nrf2 plays a key role in maintaining oxidation-reduction (redox) homeostasis by inducing the expression of multiple genes associated with antioxidant defense [15]. A highly conserved amino-terminal Neh2 domain of the Nrf2 [16] can bind to Kelch-like ECH-associated protein1 (Keap1). Under normal physiological conditions, Keap1 binds to Nrf2 and initiates its further degradation by ubiquitination or proteasome pathways [17]. Keap1/nrf2 was reported to involve in diverse aspects of cancer, such as angiogenesis [18] and drug resistance [19]. Studies show that in lung adenocarcinoma, miR-141-3p inhibits Keap1 mRNA translation and positively regulates Nrf2 activity [20]. Previous studies [12,21] demonstrated that cancer cells induced macrophages M2 phenotype transformation through Nrf2 activation. TAM secretes VEGF to promote cancer cell migration by Nrf2-induced epithelial–mesenchymal transition. These findings not only explain the cancer cell and macrophage interaction to contribute to tumor progression but also provides comprehensive insights into the role of Nrf2 in TAM formation and tumor metastasis [12]. Nrf2 overexpression was reported to be positively associated with OC progression [22], and also contributes to the chemoresistance and angiogenesis in OC [23]. This study will explore whether miR-141-3p can regulate NRF2 expression by targeting Keap1 to promote M2 transformation in OC *in vivo* and *in vitro*, thus providing a theoretical basis for the pathogenesis and treatment strategy of OC through reducing M2 macrophages.

## 2 Methods

### 2.1 Patients and tumor tissues

A total of 28 surgery resected OC samples from our hospital were selected. The patients were diagnosed with epithelial OC or malignant ovarian germ cell tumors by postoperative pathology and confirmed not receiving radiotherapy or chemotherapy before operation. Patients with other malignant tumors or autoimmune diseases were excluded. All the samples were stored in –80°C refrigerator after collection. Another 28 matching non-cancer specimens were selected as control. All subjects signed informed consent voluntarily for using their sample and clinical data, and this study was approved by the Ethics Committee of the Fourth Hospital of Hebei Medical University.

### 2.2 GSE dataset collection

The microarray data from the GEO database (http://www.ncbi.nlm.nih.gov/gds/) were retrieved to verify our findings. The detailed screening criteria included homo sapiens, OC, and expression profiling. The selected dataset should include OC sample and non-carcinomatous sample, and each group contained more than ten samples. Finally, the GSE81873 dataset were obtained, including primary tumor tissue of 19 advanced epithelial OC patients and 13 samples from benign ovarian masses were assayed. No ethical approval or informed consent was required due to the public availability of data.

### 2.3 Cell culture

Human endometrial epithelial cells were purchased from Shanghai Cell Bank of the Chinese Academy of Sciences and cultured in DMEM medium supplemented with 10% fetal bovine serum (FBS). OC cell lines, A2780 and SKOV3, as well as macrophage cell THP-1, (all purchased from Cell Center of Peking Union Medical College) were cultured in RPMI 1640 medium (Hyclone Corporation, USA) supplied with 10% FBS (PAN, Germany) and cyanin-streptomycin (100 U/ml and 0.1 mg/ml, Beyotime, Shanghai, China), and the cells were cultured at 37°C, 5% CO2 incubator. When the cells reached 70–80% fusion, 0.5% trypsin was used for cell digestion and passage.

### 2.4 Co-culture of SKOV3 and THP-1

Human monocytic cell line THP-1 (1 × 10^5^/well) were incubated with 100 ng/ml PMA for 48 h to achieve their differentiation towards macrophage, termed as M0 macrophages. Then, transwell inserts were placed into a six-well plate for co-culture. M0 macrophages were seeded into the lower chamber and SKOV3 or A2780 cells with various treatment were seeded into the upper chamber. 24 h after incubation, cell proliferation was determined by CCK8 assay, and M2 polarization status of macrophages were determined by flow cytometry using M2 biomarker CD206 (BioLegend, CA, USA). The IL-10, Arg1, iNOS, and TGF-β mRNA levels in macrophages were also measured by Quantitative Real-time PCR (qRT-PCR) assay.

### 2.5 Flow cytometry

Polarization of macrophages were collected for flow cytometry. The cells were maintained with PE-labeled CD206 (BioLegend) for 30 min at room temperature. Next the stained cells were analyzed using a BD Accuri™ C6 cytometer (BD Biosciences).

### 2.6 Cell transfection

Cells (1 × 10^6^/well) were inoculated on six-well plates. When 80% confluence was achieved, 100 nmol/L miR-141-3p inhibitor plasmid and negative control (NC) (designed and synthesized by GenePharma, Shanghai, China) were transfected into OC cells according to the instruction of Lipofectamine 2000 regent [24]. The transfection efficiency was determined by qRT-PCR.

### 2.7 qRT-PCR

Total RNA was extracted from the OC cells using Trizol reagent (Invitrogen, CA, USA). Reverse transcription and qRT-PCR were performed according to the instruction of the Prime script RT Reagent Kit (Takara, Tokyo, Japan) and SYBR PrimeScript miRNA RT-PCR Kit (Takara, Japan). U6 and β-actin were used as an internal reference, and all the involved primer sequences are shown in Table 1.

**Table 1:** qRT-PCR primer sequences

Gene	Sequence
miR-141-3p	F: 5′-GCGGAAAGAGGCCCCG-3′
	R: 5′-AGTGCAGGGTCCGAGGTATT-3′
U6	F: 5′-GAGGGCCTATTTCCCATGATT-3′
	R: 5′-TAATTAGAATTAATTTGACT-3′
IL-10	F: 5′-TCTCCGAGATGCCTTCAGCAGA-3′
	R: 5′-TCAGACAAGGCTTGGCAACCCA-3′
Arg1	F: 5′-TCATCTGGGTGGATGCTCACAC-3′
	R: 5′-GAGAATCCTGGCACATCGGGAA-3′
iNOS	F: 5′-CAGCGGGATGACTTTCCAA-3′
	R: 5′-AGGCAAGATTTGGACCTGCA-3′
TGF-α	F: 5′-CAGATTCCCACACTCAGTT-3′
	R: 5′-TACCCAGAATGGCAGACACA-3′
β-actin	F: 5′-CACCATTGGCAATGAGCGGTTC-3′
	R: 5′-AGGTCTTTGCGGATGTCCACGT-3′

### 2.8 CCK-8 assay

Cells at logarithmic growth with concentration of 5 × 10^4^/well (100 μl) were inoculated in a 96-well culture plate. The proliferation of cells was measured at 24, 48, and 72 h according to the instructions of the CCK-8 Kit (Beyotime, Beijing, China). The absorbance value (OD value) at 450 nm wavelength was measured by using a Multiskan MK3 microplate reader (Thermo Fisher Scientific, CA, USA) and experiments were repeated three times.

### 2.9 Wound-healing assay

OC cells with a concentration of 5 × 10^4^ cells/well were inoculated into a six-well plate. After the cells adhered to the culture medium, a sterilized two-milliliter pipette tip was used to generate wounding across the cell monolayer, and the debris was washed with PBS twice. The cells were observed with an inverted fluorescence microscope at 0, 24, and 48 h after treatment. The scratch width was calculated by ImageJ software (NIH, USA).

### 2.10 Transwell assay

SKOV3 and A2780 cells were diluted with a cell density of 2 × 10^5^ cells/ml. 200 μl of cells were loaded into the upper chamber of a 24-well culture insert (Corning, USA), the chamber was preloaded with Matrigel (BD Biosciences, USA), and 1 ml RPMI-1640 culture medium was supplemented. The lower chamber was cultured for 24 h with 600 μl of RPMI-1640 medium containing 10% FBS. The cells which migrated into the bottom surface were fixed in 4% paraformaldehyde for 20 mins and dried at room temperature (25°C), then dyed with crystal violet for 20 mins. The invasion of cells was observed under a light microscope, and the average number of cells passing through the Matrigel matrix was calculated. The experiment was repeated three times.

### 2.11 Dual-luciferase reporter assay

The NC plasmid and miR-141-3p mimics were co-transfected with the 3′-untranslated regions (UTR) reporter vector with wild type Keap1 3′-UTR sequence (pGL3 Keap1 3′-UTR-WT) and mutant KEAP1 (pGL3Keap1-MT, Promega, USA) reporter vector. After being transfected for 48 h, the supernatant of cells was absorbed and washed with cold PBS. Lysis buffer was added to lysate cells. The lysed cells were collected. The luciferase activity of cell supernatant was detected by luciferase Kit (cat#: RG009, Beyotime). The relative luciferase activity of each group was calculated.

### 2.12 *In vivo* analysis of the M2 polarization of macrophage in xenograft mice

Six-week-old female C57BL/6J mice (purchased from the animal experiment center of Hebei Medical University) with weights of 20–23 g were used to establish a tumor model with six in each group. A total of 0.2 ml cell suspension with a concentration of 2 × l0^6^ ml OC cells, stably transfected with plasmid containing miRNA-NC and miR-141-3p inhibitor, were subcutaneously inoculated into the back of the nude mice. Three weeks later, the mice were then euthanized for excision of the tumors, which were weighed, fixed, and paraffin embedded. During this period, the tumor volume was measured using a vernier caliper and calculated based on the formula: (length (mm) × width (mm))/2. The tumor inhibition rate (%) = (tumor weight of control group – tumor weight of treatment group)/tumor weight of control group × 100%. Next the expression patterns of macrophage marker Mac-2 and M2 surface molecule CD206 were determined using immunohistochemistry.

### 2.13 Western blot

The protocol was modified based on publications [25,26]. Proteins of cells were lysed and collected with RIPA lysis solution. Protein concentration was determined by the BCA method. The protein samples of each group were loaded in equal quantities and transferred to the polyvinylidene fluoride membrane (BD Biosciences, USA) after electrophoresis. The membrane was blocked with QuickBlock™ blocking buffer (Beyotime) at room temperature and incubated overnight with a specific primary antibody for Keap1, Nrf2, HO-1, and β-actin (#ab227828, #ab137550, # ab13243, and #ab115777, Abcam, UK) at 4°C. TBST was used for a full washing, and the corresponding secondary antibody was added and incubated at room temperature for 1 h. After washing, ECL luminescent solution (Millipore, USA) was dropped to visualize protein bands. β-actin served as the internal reference.

### 2.14 Rescue assay

Nrf2 activator-2 (compound O15, HY-145879) was purchased from Med chem express (MCE, CA, USA), and this compound (5.0 μM) was added into the cell culture medium of SKOV3 followed by miR-141-3p inhibitor treatment. After 48 h, the cells were harvested for subsequent assays.

### 2.15 Statistical analysis

SPSS 20.0 software was used for statistical processing. All data were expressed as mean value ± standard deviation. An independent sample *T*-test or nonparametric test was used for quantitative data of the two groups, and one-way analysis of variance was used for inter-group comparison. *p* < 0.05 was considered statistically significant.


**Ethical approval:** This study was approved by the ethics committee of Fourth Hospital of Hebei Medical University for tissue analysis and data sharing. The animal study and protocols were approved by the animal ethics and welfare committee of Fourth Hospital of Hebei Medical University. All experiments are reported following the ARRIVE guideline.

## 3 Results

### 3.1 miR-141-3p is upregulated in ovarian tumor tissues and cells

**Figure 1:**
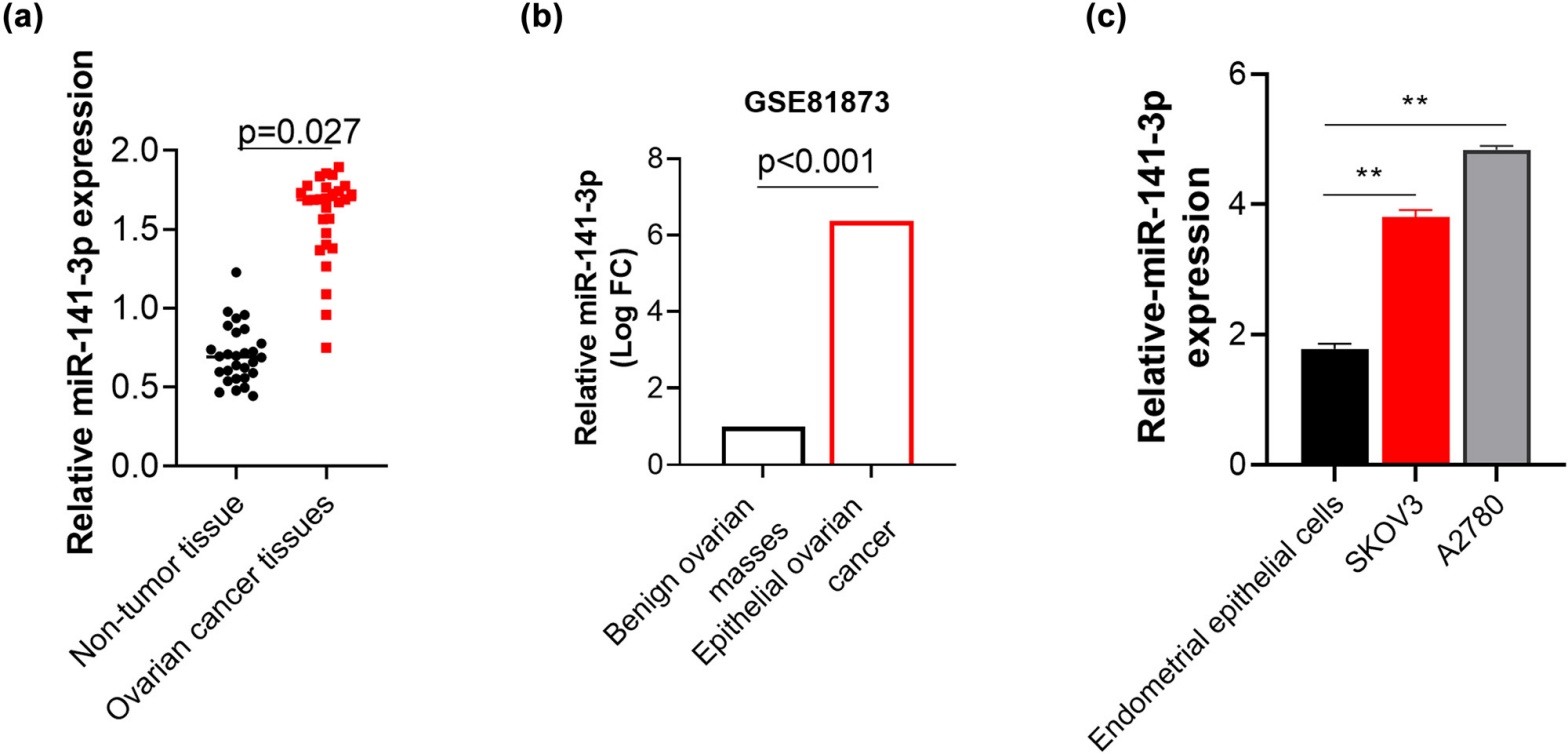
Expression of miR-141-3p in OC tissues and ovarian cells. (a) miR-141-3p expression in 28 collected paired tissues; (b) miR-141-3p expression in GSE81873 dataset, including 13 benign tissues and 19 epithelial OC tissues. The differential expression was analyzed by GEO2R, an online analysis tool of GEO and demonstrated as Log of fold change (Log FC); and (c) miR-141-3p expression in ovarian cells and normal ovarian cells. ^**^ *p* < 0.01.

In this study, 28 ovarian tissues and non-tumor ovary tissues with age matching were collected to analyze the expression of miR-141-3p. Results showed that miR-141-3p was upregulated in ovarian tissues when compared with non-tumor tissues (Figure 1a). The median Ct value of miR-141-3p was used as threshold to distinguish the higher or lower expression level in OC tissues, then the distribution of miR-141-3p expression among all the clinical parameters is shown in Table 2. The expression of miR-141-3p was significantly associated with high clinical stages and lymph node metastasis (*p* < 0.05, Table 2). Furthermore, the analyzed results of the GSE81873 dataset also support our conclusions (Figure 1b). The expression level of miR-141-3p in two OC cell lines, A2780 cells and SKOV3 cells, was significantly higher than that in human ovarian surface epithelial cell line (HOSEpiC) (Figure 1c), which suggested that miR-141-3p was over-expressed in OC, which possibly was associated with malignant behavior of OC.

**Table 2:** Expression of miR-141-3p and clinical pathology in ovarian tumor tissues

		*N*	High (*N*)	Low (*N*)	*p*
Age (year)	≤60	11	6	5	0.70
	>60	17	8	9	
Menopause	Yes	18	10	8	0.82
	No	10	6	4	
Pathological type	Epithelial OC	15	13	2	0.372
	Malignant ovarian germ cell tumors	13	9	4	
Clinical stage	Ⅰ–Ⅱ	20	6	13	0.005
	Ⅲ–Ⅳ	8	8	1	
Histological grading	Serous	21	10	11	0.378
	Mucinous	7	4	3	
Lymph node metastasis	No	21	8	13	0.029
	Yes	7	6	1	

### 3.2 Downregulated miR-141-3p inhibits proliferation and invasion of ovarian cells

**Figure 2:**
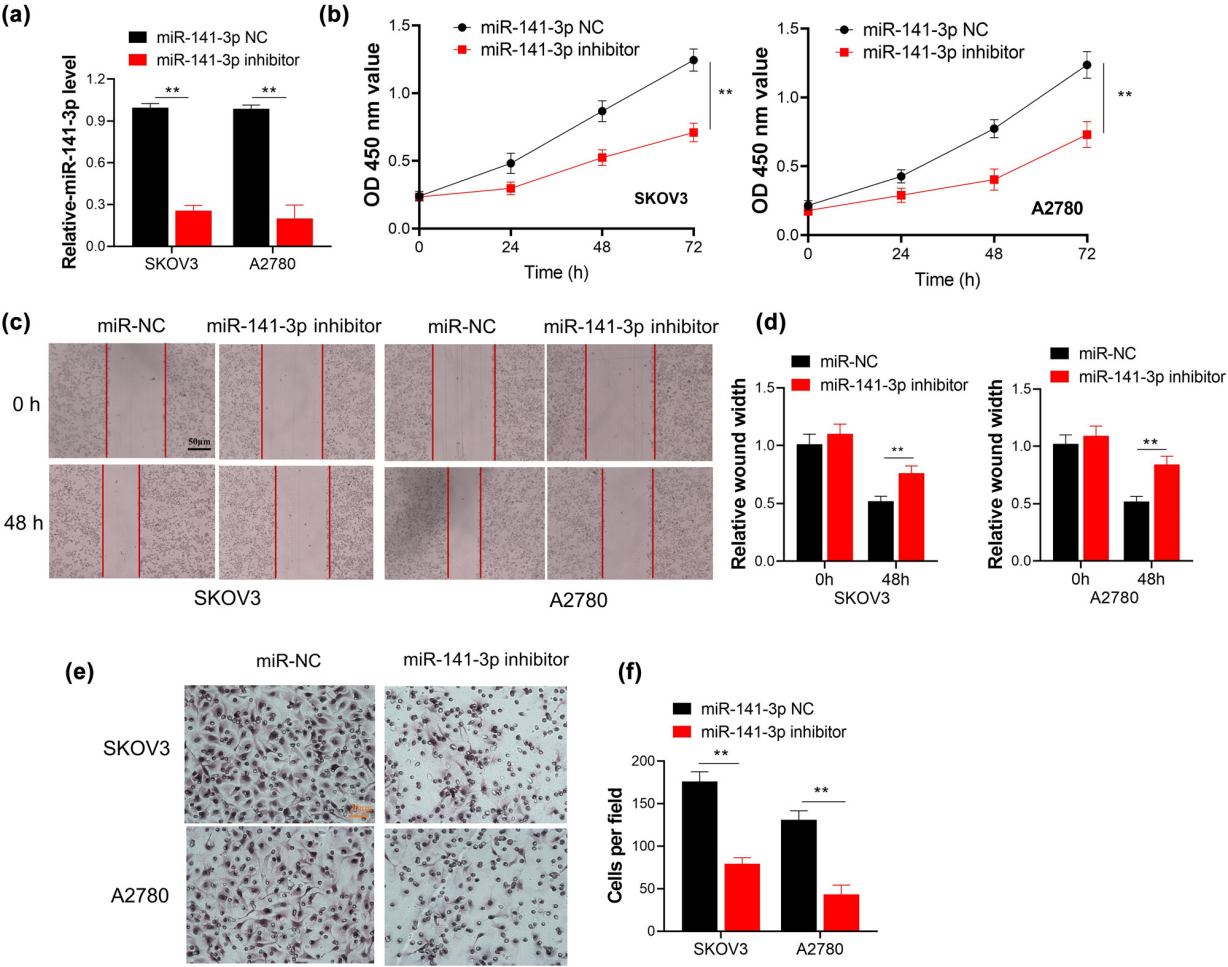
Downregulation of miR-141-3p inhibits proliferation and invasion of SKOV3 and A2780 cells. (a) The transfection efficiency in A2780 and SKOV3 cells measured by qRT-PCR; (b) cell proliferation in A2780 and SKOV3 cells tested by CCT-8 assay; (c) a representative image of ovarian cells migration characterized by wound-healing assay; scale bar, 50 μM; (d) comparisons of wound-healing width between two groups performed by paired *t*-test. (e and f) transwell assay was carried out to test the invasion of SKOV3 cells. The cells number per field was analyzed by Image J. scale bar, 20 μM; ^**^ *p* < 0.01.

To further explore the role of miR-141-3p in OC, miR-141-3p NC or miR-141-3p inhibitor was transfected into SKOV3 and A2780 cells. The transfection efficiency for miR-141-3p downregulation was confirmed by qRT-PCR (Figure 2a). The proliferation of two ovarian cells was inhibited after transfected with miR-141-3p inhibitor (Figure 2b). Furthermore, the migration ability of ovarian cells decreased due to inhibition of miR-141-3p (Figure 2c and d). Subsequent transwell assays with Matrigel additionally confirmed that miR-141-3p inhibition impaired invasion capacity of OC cells (Figure 2e and f). Thus, these data supported that miR-141-3p promoted proliferation, migration, and invasion of OC cells.

### 3.3 MiR-141-3p promotes M2 polarization *in vitro*

**Figure 3:**
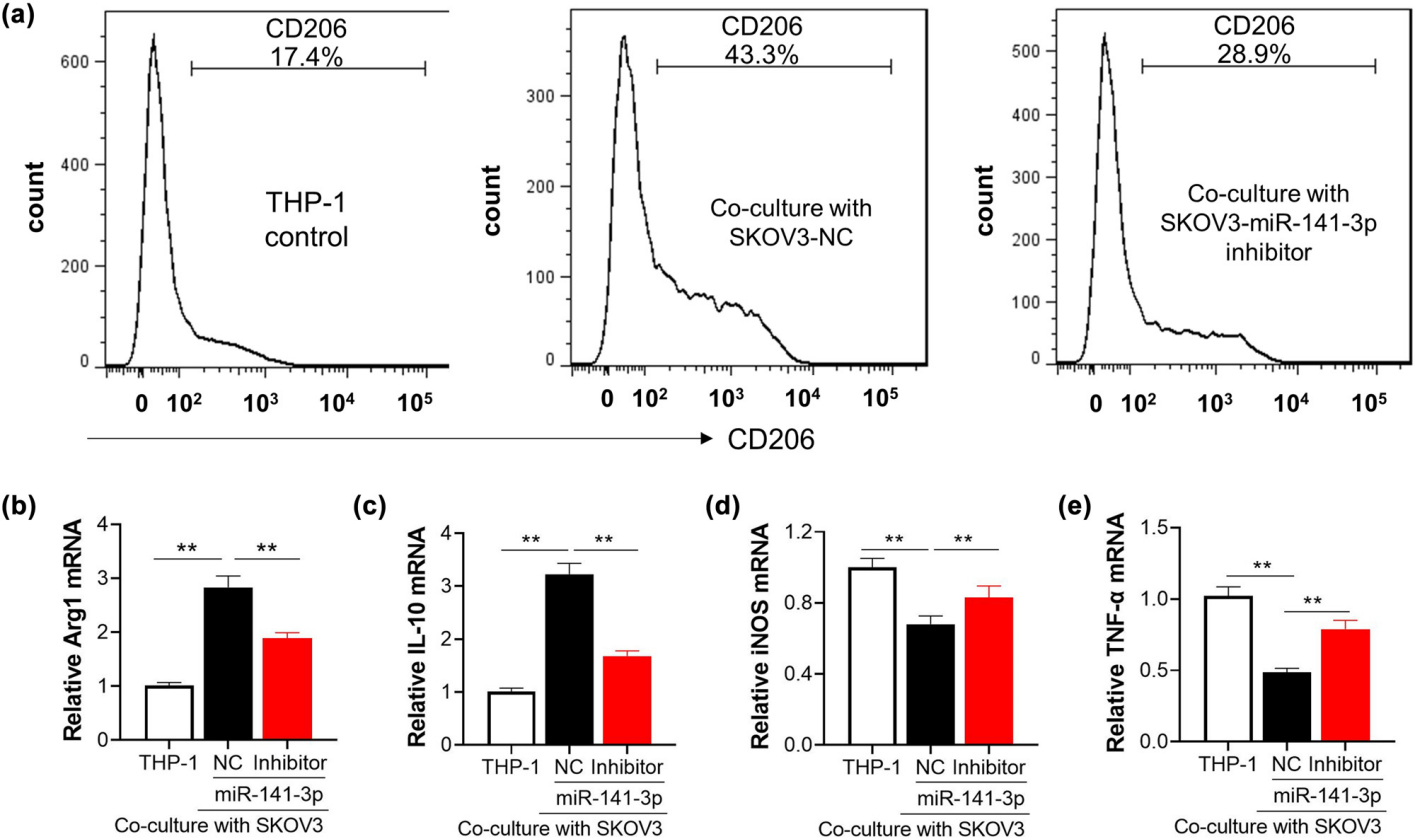
MiR-141-3p promotes M2 polarization *in vitro*. M0 THP-1 macrophage was co-cultured with SKOV3 cells transfected with miR-141-3p inhibitor for 48 h. (a) M2 biomarker CD206 were detected by flow cytometry. (b–e) qRT-PCR was performed to measure the mRNA level of specific molecules (Arg1, IL-10, iNOS, and TNF-α), respectively corresponding to M1 and M2. ^**^ *p* < 0.01.

SKOV3 cells were selected to investigate the influence of miR-141-3p on THP-1 macrophage polarization due to relatively higher expression of miR-141-3p. The plasmids containing miR-141-3p NC or miR-141-3p inhibitor were transfected into SKOV3 cells, respectively. Then, different groups of SKOV3 cells were co-cultured with differentiated M0 type macrophages. The surface markers of M2 were detected by flow cytometry. The results showed that M0 type macrophages co-cultured with SKOV3 (NC) remarkedly upregulated the expression of CD206 in M0 compared with the normal cultured control cells, while this phenomenon was reversed by miR-141-3p inhibitor transfection in ovarian cells (Figure 3a). Consistently, M2 specific molecules including Arg1 and IL-10 were declined by miR-141-3p inhibition. On the other hand, M1 specific molecules including iNOS and TNF-α were elevated when M0 macrophages were co-cultured with SKOV3-miR-141-3p inhibitor compared with SKOV3-NC (Figure 3b–e). These results demonstrated that miR-141-3p contributed to the M2 polarization of macrophages *in vitro*, and miR-141-3p inhibition could attenuate M0 polarization towards M2 like phenotype.

### 3.4 MiR-141-3p downregulation prevented growth of OC cells and reduced M2-like macrophage polarization *in vivo*

**Figure 4:**
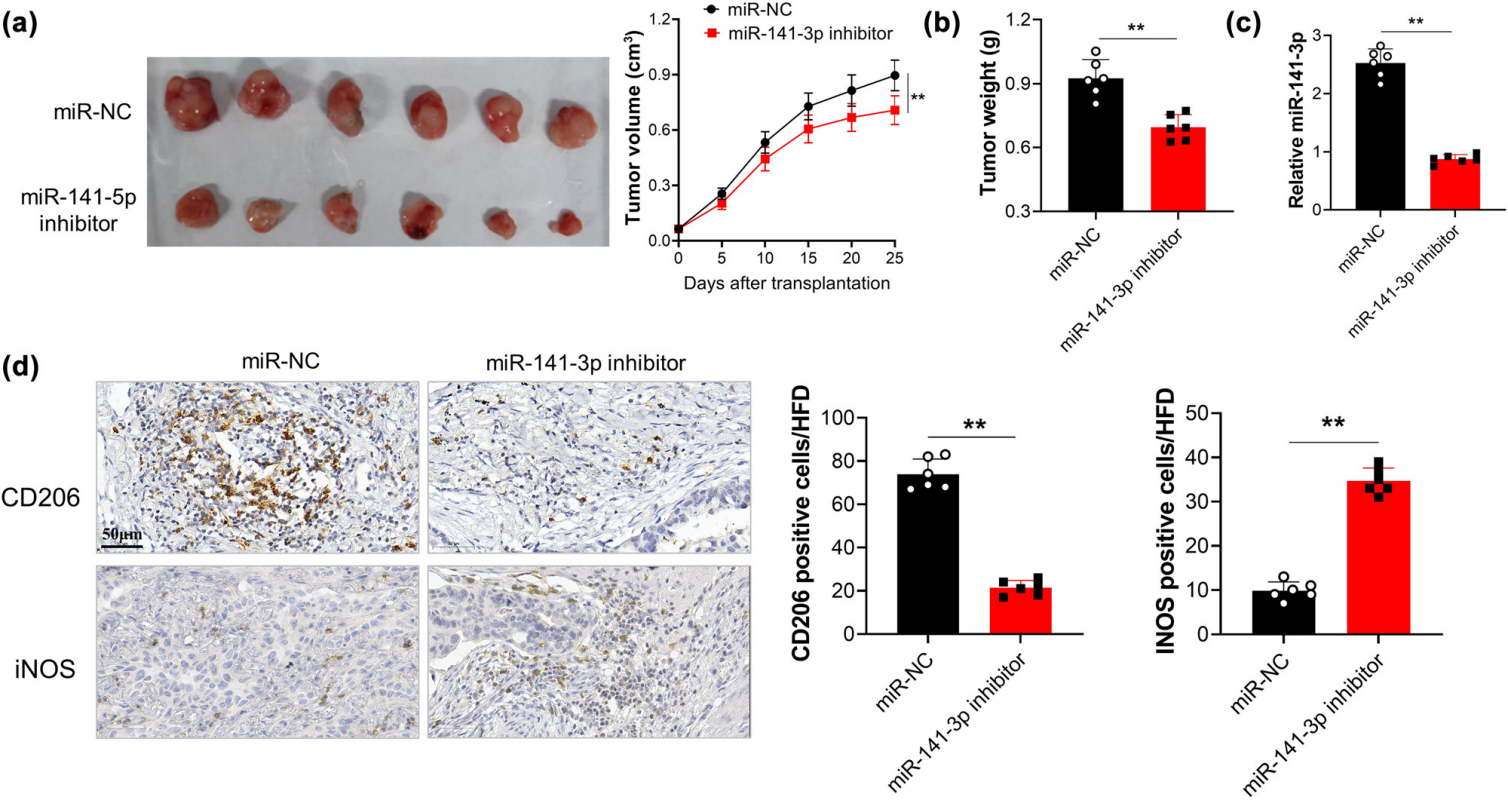
The effect of miR-141-3p on OC cell development and M2 polarization were analyzed *in vivo*. (a and b) tumor volume and weight were recorded. (c) miR-141-3p expression within tumor tissue of mice was measured by qRT-PCR. (d) M1 marker iNOS and M2 marker CD206 determined by immunohistochemistry. ^**^ *p* < 0.01; Bars, 50 μm.

SKOV3 cells stably transfected with miR-141-3p inhibitor or NC were subcutaneously injected into the back of female nude mice, to investigate whether miR-141-3p affected OC xenograft *in vivo*. Mice xenografted with SKOV3 with miR-141-3p inhibitor exhibited reduced tumor volume and weight compared with NC group (Figure 4a and b). qRT-PCR also confirmed that miR-141-3p expression was significantly lower in tumor tissues from mice of miR-141-3p inhibitor group compared with NC group (Figure 4c). Moreover, miR-141-3p inhibition remarkably reduced the M2 marker CD206 positive cells infiltration into the tumor tissues (Figure 4d), while iNOS as M1 biomarker increased instead.

### 3.5 miR-141-3p directly targeted keap1 and downregulated its expression

**Figure 5:**
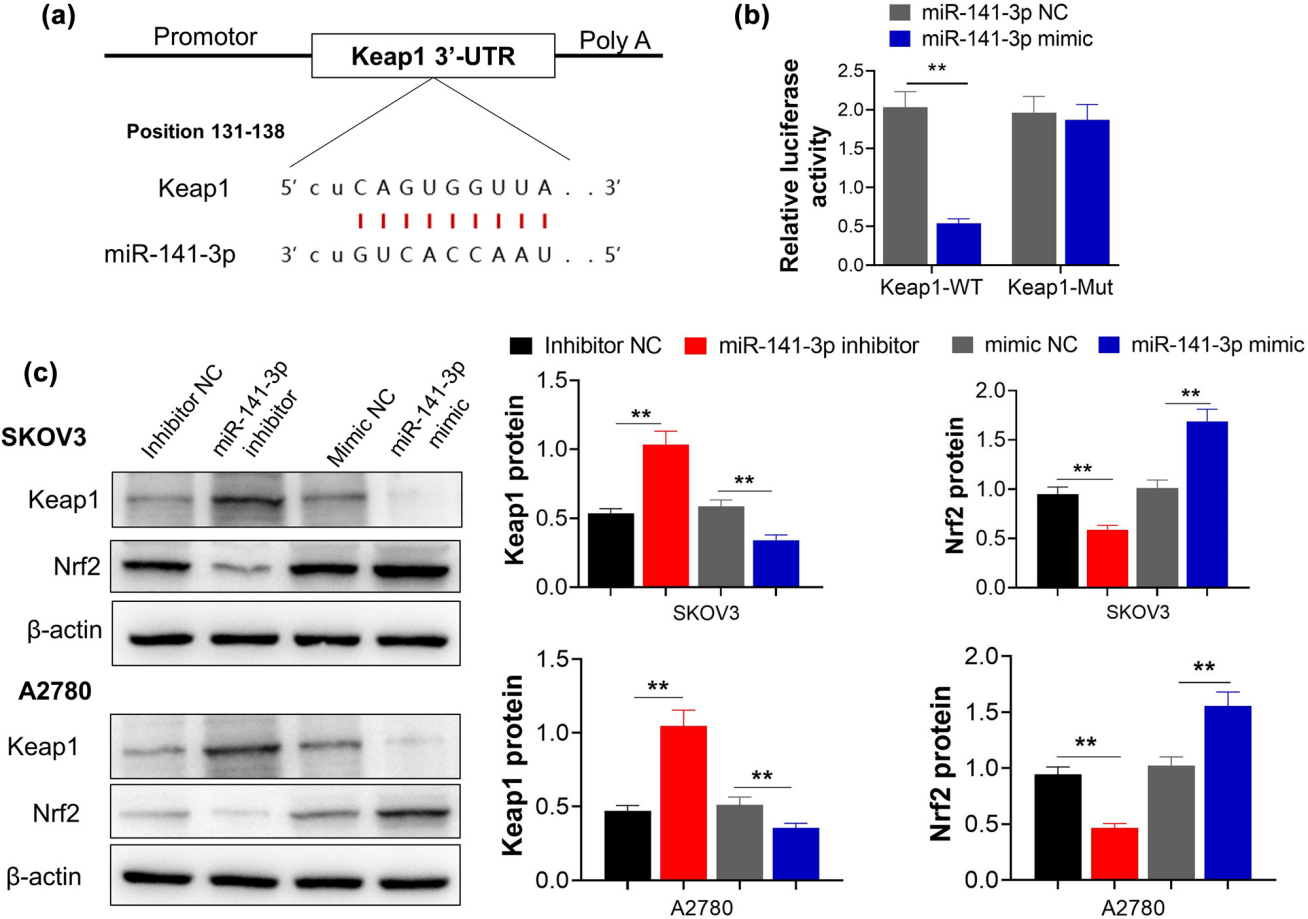
miR-141-3p directly binding to Keap1 and affects its expression. (a) the binding site of miR-141-3p to mRNA of Keap1 predicted by TargetScan; (b) dual-luciferase reporter assay result predicted the targeted binding relationship between miR-141-3p and Keap1; (c) representative western blot image and column comparison of protein expression in SKOV-3 cells with various treatment. ^**^ *p* < 0.01.

Bioinformatics method was utilized to find the possible downstream molecules of miR-141-3p in OC. The binding sites of miR-141-3p and Keap1 were predicted using TargetScan (http://www.Targetscan.Org/) gene prediction software. The dual luciferase reporter gene assay showed that the luciferase activity was significantly decreased after co-transfection of miR-141-3p mimics with pGL3 3′-UTR of Keap1-WT reporter vector (*p* < 0.05 vs Keap1-Mut reporter vector) (Figure 5a and b). To further evaluate the direct regulation of miR-141-3P on keap1, we designed the following treatment for SKOV3 and A2780: mimics NC, miR-141-3p mimics, inhibitor NC, and miR-141-3p inhibitor. After treatment for 24 h, the cellular protein levels of keap1 and Nrf2 were detected by western blot. As displayed in Figure 5c, keap1 was up-regulated when miR-141-3p was inhibited, however, this was reversed by miR-141-3p mimics; meanwhile, Nrf2 showed opposite trend. Collectively, the above data indicated that miR-141-3p could suppress keap1 to boost M2 polarization and promote tumor development.

### 3.6 miR-141-3p mimic upregulated OC proliferation and M2 polarization via Keap1/Nrf2 pathway

**Figure 6:**
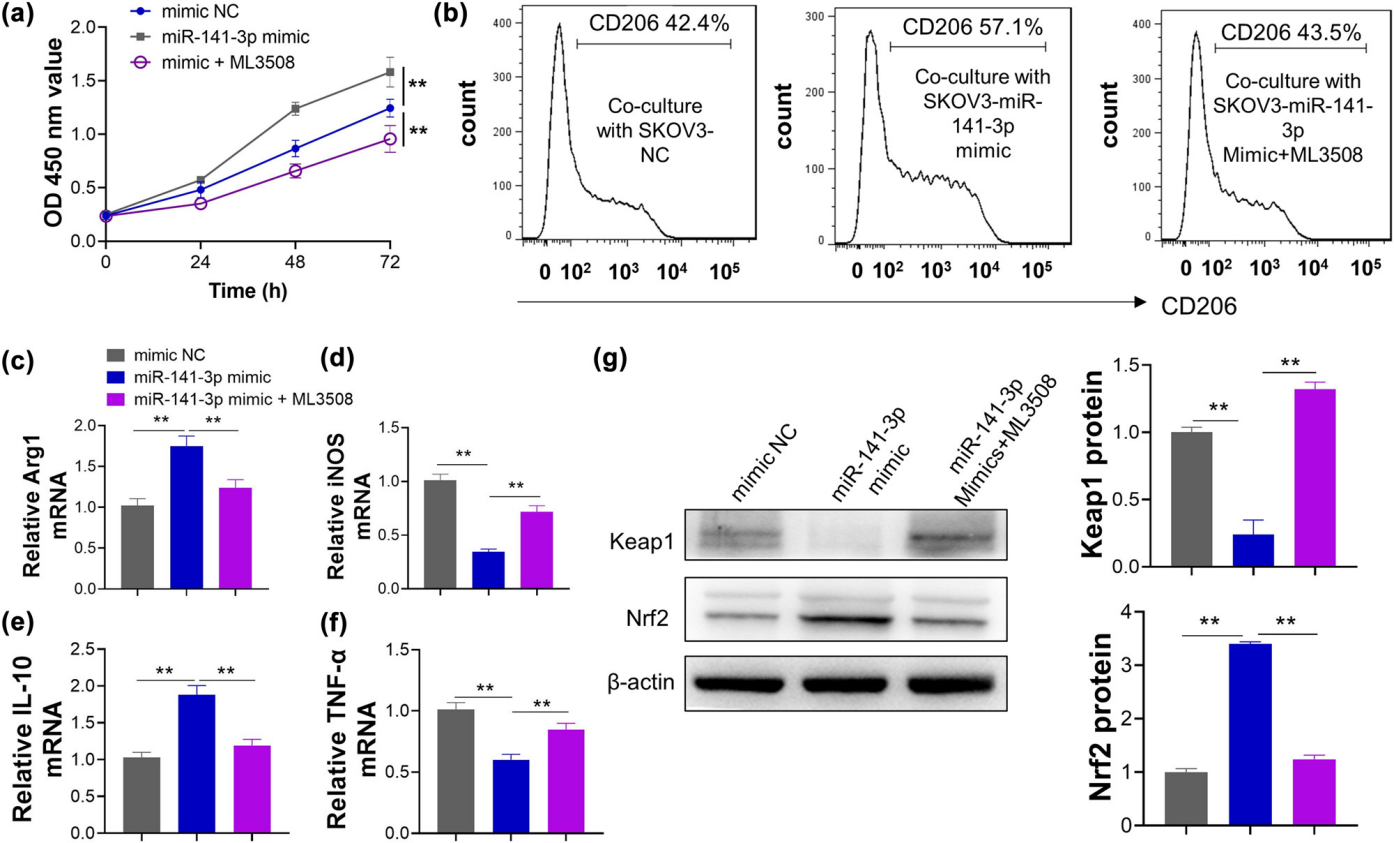
miR-141-3p promotes OC proliferation and M2 polarization via Keap1/Nrf2 pathway. (a) The miR-141-3p mimic promotes the proliferation of SKOV3 cells, while the promotional effect of miR-141-3p was abolished by Nrf2 inhibitor ML3508; (b) miR-141-3p mimic increases expression of M2-like macrophage biomarker CD206 and this effect is abolished by Nrf2 inhibitor; (c–f) The increasing mRNA levels of Arg1 and IL-10, and decreasing mRNA levels of iNOS and TNF-α caused by miR-141-3p mimic are reversed by Nrf2 inhibitor ML3508; (g) The protein levels of Keap1 and Nrf2 in SKOV3 cells after being treated by miR-141-3p NC, miR-141-3p mimic, and miR-141-3p mimic + ML3508.

To determine whether the proliferation and M2 polarization induced by miR-141-3p is dependent with direct degrading of keap1, miR-141-3p mimics and NC mimics were transiently transfected into the SKOV3. Results demonstrated that SKOV3 cells transfected with miR-141-3p mimics significantly increased proliferation of cells, but its effect was abolished by Nrf2 inhibitor ML3508 (Figure 6a). Consequently, cancer cells were employed to co-culture with M0 macrophages. As anticipated, Nrf2 inhibitor reversed the effect of miR-141-3p mimic on M2 polarization. Changes in specific biomarkers for M1 and M2-like macrophage by miR-141-3p were partly restored by Nrf2 inhibitor, which included CD206 determined by flow cytometry (Figure 6b), and iNOS, TNF-α, Arg1, and IL-10 were confirmed by qRT-PCR (Figure 6c–f). In the protein level, Nrf2 activation by miR-141-3p mimics was abolished by ML3508, which further suggested that miR-141-3p effect on OC cells was dependent on keap1/Nrf2 signaling pathway (Figure 6g).

## 4 Discussion

OC is one of the most common malignant tumors in women in the clinic. Patients with OC have a high risk of long-term recurrence and the overall survival rate is relatively low [27]. The pathogenesis of OC involves multiple genes and steps [9]. Currently, there are neither effective targeted therapeutic drugs nor markers that can predict the long-term prognosis.

miRNAs are small non-coding molecules that recognize 3′-UTR of specific mRNAs and negatively regulate mRNA abundance through translation blocking or forced degradation [20]. Our study manifested that miR-141-3p was aberrantly upregulated in OC both *in vitro* and *in vivo*, and inhibiting miR-141-3p could significantly impede ovarian cells proliferation and migration as well as tumor progression in nude mice. Clinical and experimental data fully demonstrated that M2-like TAM promotes the malignant evolution of tumors. At the initial stage of tumorigenesis, TAM created an inflammatory microenvironment and promoted cell mutation and growth. In the process of tumor deterioration and development, M2-1ike TAM secretes corresponding cytokines, promotes aberrant angiogenesis in tumors, and enhances the ability of tumor cells to migrate and invade [28]. At the tumor metastasis site, M2 like TAM modifies the microenvironment of the target tissue to better accept tumor cells. In addition, M2 like TAM promotes the implantation of tumor in target organs and subsequent continuous proliferation, and finally promotes the distal metastasis of tumor cells [29]. Hence, deep understanding of the M2 polarization and its regulation will add inspiration and value to cancer therapy. In this study, we further explored the influence of miR-141-3p on M2 polarization and found that ovarian cells treated with miR-141 inhibitor could suppress M2 related cytokines, while reinforce M1 macrophage polarization.

Long-term oxidative stress is highly correlated with the occurrence of many diseases, including OC [30]. Recently, keap1/Nrf2 has been believed to promote the occurrence and development of diseases, including OC [31]. In normal cells, appropriately upregulating the expression of Nrf2 can increase the antioxidant activity and reduce the risk of carcinogenesis. On the other hand, inhibiting the expression level of Nrf2 can inhibit the growth of tumor cells [32]. In normal physiological conditions, Keap1 exists in the cytoplasm and binds with Nrf2, which mediates Nrf2 inhibition. The Keap1-Nrf2 system is involved in the pathological process of different diseases. The disrupted binding of Keap1 to Nrf2 is an important step to shift cells into a state of oxidative, and their expression or regulation become abnormal in tumors [33,34]. Mitsuishi et al. [35] indicated that downregulation or deletion of the Keap1 gene significantly activated Nrf2. When continuously activated, Nrf2 is conducive to the growth of cancer cells. Dogan et al. [36] reported that serum levels of Nrf2 and Keap1 in bladder cancer patients were found to be higher than the control group. Nrf2, acting as “switch” for oxidative stress, might participate in the process of TAMs, which is stimulated by oxidative stress [37]. We speculate that miR-141-3p might regulate oxidative stress in tumor cells through indirect regulation of Nrf2, and this conclusion needs to be confirmed in subsequent research.

However, the role of Keap1/nrf2 in OC is unclear at present. The results of the current study showed that compared with the normal group, miR-141-3p was downregulated, the expression of Keap1 was increased, and Nrf2 was decreased, which was correlated with the enhanced invasion and migration ability of ovarian cells. Using TargetScan (http://www.targetscan.Org/), online prediction software, we predicted that miR-141-3p binds with Keap1, and dual luciferase experimental results further validate that miR-141-3p downregulates expression of Keap1. Additionally, using miR-141-3p mimic and inhibitor, keap1/nrf2 was found to be negatively regulated by miR-141-3p.

Immune microenvironments play important roles in tumor progression. Recent research demonstrated promoting Nrf2 stabilization and activation induces polarization from M1 to M2 in cancer cells HCT-116 and MCF-7 [21]. Keap1 contains stress sensors in response oxidative stress pathway [38], downregulation of Keap1 leads to Nrf2 to activate a series of downstream genes and the activation of oxidative stress, this creates an advantageous condition for TAM [39,40].

Currently, the therapeutic application of miRNA in human diseases have entered the stage of clinical trials. Once overcoming the degradation of miRNA nuclease and the adverse reaction of non-specific inflammatory immune response, miRNA can play its potential in clinical treatment [41,42]. Our study shows that miR-141-3p indirectly activates Nrf2 through binding Keap1, which enhances M2 polarization. Results of both *in vitro* and *in vivo* demonstrated that miR-141-3p inhibition could notably prevent M2 while boost M1 macrophage polarization, at least partly via regulating keap1/Nrf2. These results suggest that designing antisense oligonucleotides of miR-141-3p could regulate M2 polarization *in vivo*. Of course, our research conclusion is preliminary. The dysregulated expression of miR-141-3p in OC patients need to be analyzed in a larger clinical sample, and the specific regulatory effect of inhibiting miR-141-3p in OC needs further exploration and verification. The packing vector, administration site, and dosage of miR-141-3p inhibitor require further research and exploration.

At present, there is little clinical consensus on how to choose treatment for OC after frontline progression. Immunotherapy, chemotherapy, radiotherapy, targeted therapy, and other therapies have great potential for synergistic effects, bringing hope for improving the treatment and survival of OC. Our study provides a working basis for the study of miR-141-3p as an immune therapeutic target in OC. Further research is needed to explore the effect of regulating miR-141-3p in OC patients.

In a conclusion, our study demonstrates that miR-141-3p is upregulated in OC patients and targets Keap1 to increase the expression of Nrf2, which promotes M2 transformation and boosts the tumor progression. Inhibition of miR-141-3p prevented the activation of Keap1/Nrf2 pathway to some degree and attenuates M2 polarization, thus, suppressing OC development. miR-141-3p has a potential to be used as a clinical drug target for the treatment of solid tumors, and it can also be an effective targeted therapeutic marker that can predict the long-term prognosis for OC.
